# Radiation oncologists’ attitudes and beliefs about intensity-modulated radiation therapy and stereotactic body radiation therapy for prostate cancer

**DOI:** 10.1186/s12913-020-05656-x

**Published:** 2020-08-26

**Authors:** Bruce L. Jacobs, Megan Hamm, Flor de Abril Cameron, Jose G. Luiggi-Hernandez, Dwight E. Heron, Jeremy M. Kahn, Amber E. Barnato

**Affiliations:** 1 Department of Urology, University of Pittsburgh, 5200 Centre Avenue, Suite 209, Pittsburgh, PA 15232 USA; 2 Center for Research on Health Care, University of Pittsburgh, Pittsburgh, PA USA; 3 Qualitative Evaluation & Stakeholder Engagement Services (Qual EASE), University of Pittsburgh, Pittsburgh, PA USA; 4 Department of Psychology, Duquesne University, Pittsburgh, PA USA; 5 Department of Radiation Oncology, University of Pittsburgh, Pittsburgh, PA USA; 6 Department of Critical Care Medicine, University of Pittsburgh, Pittsburgh, PA USA; 7 Dartmouth Institute for Health Policy and Clinical Practice, Lebanon, NH USA; 8 Geisel School of Medicine at Dartmouth, Hanover, NH USA

**Keywords:** Stereotactic body radiation therapy, Intensity-modulated radiation therapy, Qualitative research, Interviews, Radiation oncologists

## Abstract

**Background:**

To better understand how radiation oncologists perceive intensity-modulated radiation therapy (IMRT) and stereotactic body radiation therapy (SBRT) for prostate cancer and how these perceptions may influence treatment decisions.

**Methods:**

We conducted semi-structured interviews of radiation oncologists between January–May, 2016. We used a purposeful sampling technique to select participants across a wide range of experience, regions, and practice types. Two trained qualitative researchers used an inductive, iterative approach to code transcripts and identify themes. We then used content analysis and thematic analysis of the coded transcripts to understand radiation oncologists’ attitudes and beliefs about IMRT and SBRT.

**Results:**

Thematic saturation was achieved after 20 interviews. Participants were affiliated with academic (*n* = 13; 65%), private (*n* = 5; 25%), and mixed (*n* = 2; 10%) practices and had a wide range of clinical experience (median 19 years; range 4–49 years). Analysis of interview transcripts revealed four general themes: 1) most radiation oncologists offered surgery, brachytherapy, IMRT, and active surveillance for low-risk patients; 2) there was no consensus on the comparative effectiveness of IMRT and SBRT; 3) key barriers to adopting SBRT included issues related to insurance, reimbursement, and practice inertia; and 4) despite these barriers, most participants envisioned SBRT use increasing over the next 5–10 years.

**Conclusions:**

In the absence of strong opinions about effectiveness, nonclinical factors influence the choice of radiation treatment. Despite a lack of consensus, most participants agreed SBRT may become a standard of care in the future.

## Background

Intensity-modulated radiation therapy (IMRT) and stereotactic body radiation therapy (SBRT) are two relatively novel radiation treatments for prostate cancer. Both these treatments demonstrate encouraging initial outcomes, including good cancer control and minimal toxicity [1, 2]. Yet these treatments exhibit varying utilization patterns [3]. When IMRT emerged around 2001, it was rapidly adopted [4]. In contrast, SBRT’s initial adoption around 2007 was more variable—generally slow with some areas of rapid uptake [3]. In addition, there is wide regional variation in the use of these two treatments, with greater use of SBRT among patients residing in urban areas and by those living in the northeast [3].

A key source of this variation may be the attitudes and beliefs of the physicians who prescribe these treatments and therefore play an essential role in their adoption. To date, at least two surveys have queried physicians about their use of IMRT or SBRT [5, 6]. These studies found that physicians using SBRT worked more frequently in academic settings and completed training more recently [6]. However, these studies did not specifically investigate why providers preferred one treatment over another. Further, they focused on the treatment of all cancers rather than just prostate cancer, and, at the time, SBRT for prostate cancer was only used by 8% of responding physicians [6]. As a result, there is a persistent knowledge gap about how physicians make decisions regarding the use of IMRT and SBRT for prostate cancer. A better understanding of the decision making process will elucidate potentially modifiable factors that policymakers and other interested stakeholders can focus their attention on to either accelerate or decelerate SBRT use depending on the latest scientific evidence and, perhaps more importantly, help refine the health care environment to maximize the chance that new prostate cancer technologies are adopted at appropriate rates.

For these reasons, we conducted semi-structured interviews of 20 radiation oncologists to better understand their attitudes and beliefs about IMRT and SBRT. We sought to use qualitative insights to develop hypotheses regarding barriers and facilitators of the adoption of new radiation technologies to offer insights into potential intervention targets for technology adoption and the provision of high value care for prostate cancer.

## Methods

### Study design

We used a qualitative description approach, which is frequently employed in the health sciences when the goal is a description or understanding of a particular phenomenon, but without describing a culture (as in ethnography), individual lived experience (as in phenomenology), or to build theory (as in grounded theory). Using qualitative interviews, this approach allowed us to inductively investigate radiation oncologists’ experiences with and perspectives of IMRT and SBRT for prostate cancer [7]. Our objective was not to quantify these attitudes, but rather to better understand what factors influence the physicians’ decision making regarding these two treatments. By using a qualitative approach, we sought to uncover new insights and novel hypotheses about this decision-making process for which little is currently known.

### Interview guide

We developed an interview guide, which was created through an iterative process and refined through 5 pilot interviews and multiple discussions with a multidisciplinary research team that included urologists, radiation oncologists, and qualitative research experts (Supplementary File). The final interview guide addresses the following domains: 1) spectrum of treatments offered for patients with low-risk disease; 2) comparative effectiveness of IMRT and SBRT; 3) barriers to adopting IMRT and/or SBRT; and 4) future treatment landscape for prostate cancer.

### Interviewee selection process

To select interviewees, we used a purposeful sampling technique [8] to identify a diverse group of radiation oncologists who treat prostate cancer. To ensure that we obtained a diverse sample, we selected candidates across a wide range of experience (i.e., years in practice), geographic regions, and types of practice (i.e., private practice, academic, mix). We identified potential interviewees by asking the previous interviewee to recommend another individual to participate, a practice known as “snowball sampling” [9]. No more than one physician was interviewed from a practice. We continued to interview subjects until we interviewed individuals from varying experience levels, geographic regions, and types of practices, and until we achieved thematic saturation, which we defined as three consecutive interviews in which no new information was generated [10]. We anticipated reaching thematic saturation after 15 to 20 interviews, as is typical in samples of homogenous informants [11, 12].

### Interviews

All interviews were conducted by a physician who treats prostate cancer. Before starting the interview process, the interviewer received coaching and performed practice interviews with non-study radiation oncologists to maximize the ability to elicit the respondent’s complete response to open-ended questions. Verbal consent was obtained prior to each interview. The interviewer conducted audio-recorded phone interviews designed to last 30 min. The “grand tour” question to begin the conversation was: *“Tell me about the treatments you generally consider for patients with low-risk prostate cancer?”* We focused on low-risk disease since active surveillance, surgery, and radiation are all reasonable options [13]. Open-ended questions were then asked about why they chose or did not choose IMRT or SBRT. Interviewers were conducted from January 2016 through May 2016.

### Analyses and rigor

All interviews were transcribed verbatim with identifiers removed. The interviewer reviewed a sample of transcripts to ensure that transcribed interviews were consistent with the recalled conversation. Two trained qualitative research specialists developed the codebook through an iterative process known as “editing” where the semi-structured interview transcripts were reviewed for important topics that emerged [14]. After the codebook was completed and agreed upon, the primary coder coded all 20 transcripts while the secondary coder coded 10 transcripts, both with the assistance of Atlas.ti software (Berlin, Germany). Kappa statistics were calculated to measure the inter-coder reliability with a result of 0.78, which Landis and Koch describe as “substantial” agreement [15]. Following the coding, the two coders performed a thematic analysis in which researchers identify topics, patterns, and ideas that were present in the data in order to identify themes that succinctly describe them [16, 17]. The themes derived by the coders were then shared with the qualitative expert and principal investigator as a form of investigator triangulation [18]. In addition to thematic analysis, constant comparative analysis was used to determine similarities and differences between the participants’ responses [19].

Our institution’s institutional review board deemed this study ethical and exempt from review.

## Results

### Participant characteristics

We conducted 20 interviews of radiation oncologists, including 19 men and 1 woman (Table 1). Physicians were affiliated with academic (*n* = 13; 65%), private (*n* = 5; 25%), and mixed (*n* = 2; 10%) practices. Physicians were evenly distributed across regions of the country and had a wide range of clinical experience (median 19 years; range 4–49 years).

**Table 1:** Characteristics of study population

Characteristics	Value
**Sex**	
Female	1 (5)
Male	19 (95)
**Practice type**	
Mixed	2 (10)
Private practice	5 (25)
Academic	13 (65)
**Geographic region**	
Northeast	5 (25)
Central	5 (25)
South	5 (25)
West	5 (25)
**Years in practice, median (range)**	19 (4–49)

Data are presented as number (%), unless otherwise stated

Of those who mentioned where radiation equipment was located, they all mentioned it resided at a hospital. Most participants mentioned that the equipment for performing IMRT could be used to perform SBRT, but some clarified that there were some differences in equipment, especially Cyberknife (Accuray Inc., Sunnyvale CA, USA) to perform SBRT. Participants received variable training for IMRT and SBRT. The majority of physicians had training in one or both of these modalities. Among those who lacked formal training, some were not trained because it was not available while they were residents while others were involved in the creation of the technique and thus had no training in it, per se. Some participants believed that formal training should be available for everyone and that clinicians should be certified, while others thought that they were relatively straightforward modalities that did not require formal training. Among those who used SBRT, most mentioned that the transition from delivering IMRT to SBRT was not difficult.

### General themes

Four general themes emerged from the qualitative analysis of the semi-structured interviews, including 1) treatment selection; 2) comparative effectiveness; 3) barriers; and 4) future treatment use. Themes and sub-themes are summarized below and relevant quotes are in Table 2.

**Table 2:** The four general themes with accompanying sub-themes and representative quotes

Themes	Sub-themes	Quotes
Treatment selection	Not applicable	(ID# 0021) “I mean usually, somebody with low risk prostate cancer … I’m going to be talking with them depending on their age and health about active surveillance versus prostatectomy versus … brachytherapy versus … external beam radiation. I honestly haven’t incorporated SBRT uh into my standard recommendation set just because it’s relatively new and I, I have zero experience with SBRT for prostate cancer.”
Comparative effectiveness	Clinical factors	(ID# 0008) “… more than 10 years ago, evaluating the feasibility of SBRT … we decided that the patients who would be offered this would be those with um … you know, low-volume, low-risk disease defined by … Gleason score would be 6, or 3 + 4 … 25% or fewer of the cores from the biopsy would be found to be involved with cancer, that PSA would be < 10, and that most importantly that the volume of the prostate would be < 60 cc. So we extracted a lot of what we learned from … our brachytherapy alone experience and applied it to SBRT. Uh … and the patient meeting those criteria, largely is the patient today offered SBRT uh … as an equivalent to IMRT or any other standard therapies.”
	Lack of randomized control trial data	(ID# 0007) “Yeah … that’s where there’s even more concerns about projections on long-term toxicity, so, SBRT has been done on individual … single institutional studies, um … these people have reported that the … control is good, toxicity is good … but there hasn’t been yet a very good, a randomized trial of SBRT versus a standard fractionated or even at least a hypofractionated treatment, so … whereas the hypofractionated treatments, there is now an ASTRO, GU ASCO, there were multiple presentations on NRG randomized trials that showed, within up to 5 years they are pretty much equivalent … with SBRT there is no such data, so with SBRT there is even more concern about am I going to hurt my patient and there’s so much uncertainty about is this really as effective and as safe as a standard fractionated treatment. So, um … in my mind, I would feel comfortable offering SBRT only on a clinical trial …”
	Preliminary evidence	(ID# 0008) “… Where we’ve seen some differences that are probably meaningful are in quality of life scores and, um, acute toxicities between the two, and generally in favor of stereotactic therapy, that the acute urethritis and cystitis that folks suffered during radiation therapy, um, happens over a compressed period of time with SBRT versus IMRT. Typically patients will begin to accrue these symptoms about 3 weeks or so into their course of IMRT and then they persist till the end, and then for some number of weeks thereafter. What we’ve observed with SBRT is that the return to baseline urinary scores usually occurs much more rapidly after the end of that treatment and the total length in which there is compromise that is measurable is far less.”
Barriers	Health insurance coverage	(ID# 0013) “… Up until very recently and with lots of effort, um, on some of our parts, insurance coverage of SBRT was a major barrier. That thankfully, um, is less of a problem than it used to be. All major Medicare contractors, Medicare administrative contractors, now in one fashion or another across the US cover SBRT, so that’s a good help. Of the private payers, Humana, Aetna, Cigna, cover SBRT, umm, Blue Cross Blue Shield is still spotty … it’s not 100% covered, but many areas of Blue Cross Blue Shield policy cover as well. So, non-coverage which, of course, is a pretty big barrier to adoption …”
	Reimbursement differences	(ID# 0025) “Yes, in the late 1990s, when IMRT came on the scene, uhm the, the uh powers at be managed this really good reimbursement for IMRT.” (ID# 0014) “… one of the barriers are, if, if we give 9 weeks of radiation versus a week of SBRT … it cuts your revenue stream and so that … I hate to say it that way, but I think that’s a barrier for some people.”
	Practice inertia	(ID# 0019) “So people who don’t want to do SBRT honestly are arguing that you need this technology, you need that piece of equipment, maybe spacers between the prostate and the rectum … but that’s just not true, I mean most of the um experience with uh, with uh prostate … is not necessarily using equipment that’s totally unique you know so you know, yeah there’s a lot of experience with the Cyberknife, but that’s, it’s just not true that you can’t use anything else.” (ID# 0028) “… I think uhm adoption of this technology into a community practice setting, uhm, uhm you know may be a little bit of a challenge, uhm, if they’re not used to [it].” (ID# 0030) “… just like what they mean on the advanced kind of complex technology, it’s usually larger centers that can uhm you know reach the critical mass of expertise to get these things off the ground.” (ID# 0028) “… so your margin of error should be smaller … cause you know if you miss one fraction of IMRT, well you’ve got 39 others or whatever to make up for it. If you’re off on one fraction of SBRT, that’s a big deal.”
Future treatment use	Not applicable	(ID# 0012) “… Ultimately, as the technology improves, as we’re able to monitor for prostate motion and be very accurate in how we deliver the dose, and … as with everything else, radiation is going to go to fewer fractions and tighter margins … but using better technology.” (ID# 0011) “Well I think there is going to be an increase in use of the stereotactic, SBRT, mainly because there’s going to be pressure form the insurance companies and government to do it cheaper. You think of how much it costs Medicare every time we treat some uh 70 year-old guy with prostate cancer with external beam radiotherapy? And, if you could show that with stereotactic at least 5-year, 10-year results don’t seem to be much different, they are going to push it, they are going to push it. I think that we are going to see economical considerations being used to push a particular type of treatment.”

#### Treatment selection

The most common treatments discussed included surgery, active surveillance, brachytherapy, and IMRT (Table 3).

**Table 3:** Distribution of responses regarding treatments considered for low-risk prostate cancer

Treatment	Participants, number (%)
Surgery	14 (70)
Active surveillance	13 (65)
Brachytherapy	13 (65)
IMRT	13 (65)
SBRT	9 (45)
Proton-beam therapy	3 (15)
3D-conformal therapy	2 (10)
Cryotherapy	2 (10)
Other treatments^a^	10 (50)

*Abbreviations*: *IMRT* intensity-modulated radiotherapy, *SBRT* stereotactic body radiation treatment

^a^Other treatments include image-guided radiation therapy (IGRT), other types of external beam radiation, hormone therapy, high-intensity focused ultrasound, and focal laser ablation

#### Comparative effectiveness

Participants discussed what they considered before determining which treatments to provide for their patients, which included the following factors:

##### Clinical factors

These factors included the patient’s prostate-specific antigen (PSA) level and Gleason score, the biopsy results, the prostate size and volume, the magnetic resonance imaging results, and whether or not there was extra-glandular involvement, patient motivation in the setting of organ-confined disease, patient age, comorbidities, and urinary function.

##### Lack of randomized control trial data

One of the factors mentioned the most about whether to offer patients IMRT or SBRT was the lack of randomized control trial data supporting SBRT as a safe and effective alternative to IMRT. Those who favored using IMRT were concerned about SBRT’s toxicity and disease control. Provided the lack of randomized control trial data, some participants did not feel comfortable treating patients with SBRT.

##### Preliminary evidence

Others felt that even though there was not a vast amount of scientific literature, there was enough for them to feel comfortable treating patients with SBRT. Some participants believed that SBRT had lower toxicity and better disease control than IMRT and other therapies. They preferred SBRT because of its shorter duration and cost-effectiveness.

#### Barriers

Barriers mentioned included health insurance coverage, reimbursement, and practice traditions (e.g., harder to adopt a new technology in a work culture that favors using traditional treatments).

##### Health insurance coverage

Although most participants raised health insurance coverage as an issue, only some mentioned IMRT not being covered by insurers, while most mentioned SBRT was not covered by insurers. A participant mentioned that SBRT’s coverage has improved over time. Most insurance companies still did not cover SBRT because of a lack of evidence in terms of its long-term effects, since it is a relatively new treatment. In a fee-for-service payment model, the financial reimbursement for SBRT could be less, which can act as a disincentive.

##### Reimbursement differences

Some participants felt that the lower reimbursement for SBRT as compared with IMRT was a barrier to its adoption.

##### Practice inertia

Some participants mentioned that the practice culture and lack of training at some institutions could negatively impact the adoption of a new technology, such as SBRT.

#### Future treatment use

Participants were asked about the landscape of treatments for low-risk prostate cancer in 5–10 years. There seemed to be consensus that technology will improve, which will allow for creating better decision-making tools in terms of patient treatment selection, such as the use of molecular diagnostics. They also mentioned that more data will be available for SBRT, including effectiveness and long-term toxicity (which is the main concern for most participants), and the cost for SBRT will be further reduced. Participants also believed that more health insurances will cover SBRT. Some participants considered that health insurance companies will begin using bundled payments to pay for these treatments. Many participants who use IMRT hope to provide SBRT in the future once there is more concrete data on its long-term effects. Most agreed that SBRT may become a standard of care for prostate cancer.

## Discussion

Radiation oncologists consider several treatments for patients with low-risk prostate cancer, most commonly active surveillance, surgery, brachytherapy, and IMRT. In regards to IMRT and SBRT specifically, most participants used IMRT, however there was a wide range of opinions regarding the comparative effectiveness of IMRT and SBRT. Barriers to adoption, which more often pertained to SBRT, included lack of data, insurance coverage, reimbursement, and training. Most participants felt that SBRT would be a standard of care in the next 5–10 years.

Although SBRT is currently not a commonly used modality, many participants felt it would become more of a standard of care in the future. This will likely hinge on the results of prospective studies with longer term follow up and the results of ongoing randomized clinical trials [20, 21]. If the findings from these studies are favorable, clinicians will have fewer concerns regarding the risks of long-term toxicity and cancer control, payers will support a treatment that costs less, and patients will prefer spending less time getting radiation [22].

In addition to the lack of long-term data and data from randomized trials, there were other factors thought to impede the adoption of SBRT, including insurance coverage and reimbursement. Some insurance companies do not cover SBRT for prostate cancer due to lack of evidence. For example, Medicare’s coverage of SBRT varies by region in the form of local coverage determinations, which represent a determination as to whether a service is reasonable and necessary [23, 24]. Medicare administrative contractors evaluate whether a claim complies with a local coverage determination policy provision in deciding whether or not to pay for it [25]. The lack of coverage for SBRT has dissipated over time, but is still an issue to some degree.

Another barrier to SBRT use pertains to reimbursement. Since SBRT is delivered in five sessions instead of the traditional 40–45 sessions, reimbursement is often lower for SBRT in a fee-for-service environment. However, as alternative payment models such as bundled payments gain traction, there will be a greater incentive to deliver SBRT since it is less expensive.

In contemplating the various barriers to SBRT use, it is helpful to consider the Technology Acceptance Model, developed by Davis and colleagues [26]. This model, which was designed to provide an explanation of the determinants of computer acceptance in the 1980’s, focuses on the impact of external factors on internal beliefs, attitudes, and intentions; two beliefs in particular that are relevant to behavior are *“perceived ease of use”* and *“perceived usefulness”* [26]. Many of the participants’ comments regarding SBRT pertain to these two beliefs. For example, opinions regarding the need for additional training, the ability to use the same or different equipment, and insurance coverage relate to perceived ease of use whereas opinions pertaining to data, toxicity, and clinical effectiveness relate to perceived usefulness.

There was a wide range of opinions regarding the comparative effectiveness of IMRT and SBRT, which suggests that the existing evidence does not clearly support one treatment over the other. This highlights the importance of getting patients enrolled in clinical trials and registries to generate more data in a timely manner. Some policies encourage this practice by requiring enrollment to qualify for insurance coverage. For example, some local coverage determinations will only endorse SBRT if patients enroll in a clinical trial or registry [27]. Such a registry currently exists in Florida—the Multi-Institutional Registry for Prostate Cancer Radiosurgery (NCT01226004) [28]. Coverage with evidence development represents another policy that encourages enrollment by only offering coverage if patients participate in research (i.e., a clinical trial or registry) [29]. This generates evidence to help determine whether a treatment has a net benefit [30].

In discussing the treatment selection process, participants did not mention a lot about patient goals or preferences. Admittedly, this was not explicitly asked about in the interviews. Nonetheless, given the variety of acceptable treatment options for low-risk prostate cancer, an opportunity for shared decision-making among providers and patients exists. Providers generally discuss risk and benefits of treatments well, but infrequently engage patients in shared-decision making processes or have patient preferences guide the treatment plan [31].

Our findings should be interpreted in the context of several limitations. First, opinions are based on interviews with 20 radiation oncologists, which could limit generalizability. However, we purposefully selected participants from all regions of the country, from different types of practices, and among physicians with a wide range of experience. Further, we followed rigorous qualitative methodologies that required interviewing participants until we reached thematic saturation, which is typically achieved by 15–20 interviews [12]. Second, the majority of participants interviewed were male (95%). This is in part due to the low proportion of women in the radiation oncology workforce (29%) [32]. In designing the study, we did not seek to purposefully select participants based on gender since we felt attitudes towards IMRT and SBRT were unlikely to differ based on gender. Third, these interviews occurred in 2016 and more evidence has accumulated since then supporting hypofractionated radiation [33]. Nonetheless, SBRT is still sparingly used and, in the absence of randomized controlled data, these views are still very relevant today.

## Conclusions

This study is the first to our knowledge to use qualitative analyses to examine the attitudes and beliefs of radiation oncologists about different prostate cancer treatment modalities, namely IMRT and SBRT. This uncovered several knowledge gaps not addressable with traditional quantitative research methods. There were a wide range of opinions regarding the barriers to adopting SBRT, including the need for specific training, and the comparative effectiveness of IMRT and SBRT, highlighting the lack of clarity with existing data and the importance of enrolling patients in existing registries or ongoing clinical trials. Along these lines, most participants are optimistic that SBRT will be a standard of care in the next 5–10 years once longer term data matures.

## Supplementary information

**Additional file 1:** Interview Guide.

## Data Availability

The de-identified datasets used and/or analyzed during the current study are available from the corresponding author on reasonable request. The data are stored at our institution in a secure environment with multiple firewalls and physical security measures.

## Abbreviations

IMRT: Intensity-modulated radiation therapy
SBRT: Stereotactic body radiation therapy
