# Supplementary material for: Radiation oncologists’ attitudes and beliefs about intensity-modulated radiation therapy and stereotactic body radiation therapy for prostate cancer
Source: BMC Health Serv Res. 2020 Aug 26;20:796. doi: 10.1186/s12913-020-05656-x (PMC7449079; doi:10.1186/s12913-020-05656-x)
Supplement: Supplementary file 1 — Additional file 1: Interview Guide. [file 12913_2020_5656_MOESM1_ESM.doc]

**Supplementary File.** Interview Guide^*^

*^*^Note: As is customary with semi-structured interview guides, these are guidelines for conducting the interview. The exact direction of the interview will vary, depending upon the conversation. Of particular importance throughout is to understand the mental models that physicians bring to managing patients with localized prostate cancer.*

**Request permission for audio-taping**

Are you okay if our interview is recorded? Recordings will be de-identified by removing all names and will be deleted from the recorder as soon as they are downloaded.

**Interview guidelines**

When I ask questions, I will not voice any opinions about SBRT and IMRT. To avoid making any assumptions about what you mean by certain responses, I may probe further to ask for explanations.

**Begin digital recorder:**

- May I confirm your permission to audio-tape this interview?
- Tell me about the treatments you generally consider for patients with low-risk prostate cancer?

**Probes:**

- What is it about treatment X or treatment Y that you like?
- How strong is the clinical evidence for treatment X?
- How strong is the clinical evidence for the other treatment (IMRT or SBRT)?
- Do you have any concerns about the long-term (e.g. 10-15 year) toxicity of IMRT or SBRT?
- Are there any significant barriers to using IMRT and/or SBRT? If so, what?
- Does your hospital or treatment facility have the capability to deliver (IMRT or SBRT)?
- Are you aware of any policies that either facilitate or interfere with the use of IMRT or SBRT?
- Some people are concerned that finances drive the use of some advanced technologies over others. What are your thoughts on this as it pertains to IMRT and SBRT?
- Do finances factor into why people might consider treatment X or Y?
- What do you see the landscape of treatment looking like in 5-10 years?
- How long have you been in practice?
- Did you need specific training for using IMRT and/or SBRT? What training did you have?
- What type of practice are you in (academic, private practice, mix)?
- Is there anything else you would like to discussion related to the use of IMRT or SBRT?
- Can you recommend any radiation oncology colleagues from other institutions who may be interested in talking?
